# Effects of Dexmedetomidine Use on the Renal Function of Elderly Patients Undergoing Laparoscopic Surgery: A Systematic Review and Meta-Analysis

**DOI:** 10.7759/cureus.90163

**Published:** 2025-08-15

**Authors:** Yuri Nicolay Kretchetoff, Pedro Emmanuel A Brasil

**Affiliations:** 1 Section of Anesthesiology, Hospital Central do Exército, Rio de Janeiro, BRA; 2 Instituto Nacional de Infectologia Evandro Chagas, Fundação Oswaldo Cruz, Rio de Janeiro, BRA

**Keywords:** acute renal injury, dexmedetomidine, elderly, laparoscopic surgery, length of stay

## Abstract

Postoperative acute renal injury (ARI) is a common complication found in patients after laparoscopic surgery. This study aims to review the literature systematically to check the effect of dexmedetomidine (DEX) on ARI. A systematic review was conducted, encompassing abstracts published from January 2016 to August 2024, focusing on patients aged 60 years or older undergoing laparoscopic surgery. The focus of this study was on dexmedetomidine, which was used alone or with other anesthetics during the time around surgery, at any dose. The comparator of interest was a placebo or any perioperative medication used for the same purpose. ARI, urinary output, and length of stay (LOS) were the outcomes of interest. The studies of interest were clinical trials. Remote database searches were performed at PubMed, SCOPUS, Web of Science, Embase, and Cochrane, as well as full manuscript bibliographies. The risk of bias was assessed with Cochrane's RoB2 and result confidence was assessed with Grading of Recommendations Assessment, Development and Evaluation (GRADE). We analyzed 13 studies. A consistent but not significant nephroprotective effect of DEX was observed. A neutral effect was observed in urinary output and LOS, and there is evidence of reporting bias for LOS. The confidence in these findings is either poor or moderate. The use of DEX in the elderly population undergoing laparoscopy surgeries did not reduce the LOS or urinary output, but there is moderate confidence that it has a potential nephroprotective effect.

## Introduction and background

Laparoscopic surgery in elderly patients has more complications than in younger populations. Studies report overall complication rates ranging from 17.9% to 23.68% in older patients [1], depending on advanced age, diagnostic criteria, vasopressors, mechanical ventilation, etc [1]. Minor changes in creatinine and urea after surgery may increase the length of hospital stay (LOS), mortality, the risk of chronic kidney disease (CKD), and the need for hemodialysis [2,3].

The etiology of laparoscopic perioperative acute renal injury (ARI) is complex. ARI can be triggered mainly by hypoperfusion, but inflammation and a neuroendocrine response to surgical stimulus may act as additional mechanisms [2]. In the inflammatory process, prostaglandins reduce afferent arteriolar resistance, maintaining glomerular blood flow, activating the renin-angiotensin-aldosterone system, and releasing angiotensin II. This results in efferent arteriolar vasoconstriction and maintenance of adequate glomerular pressure in states of renal hypoperfusion. If this state persists or hypovolemia is greater than the capacity to maintain renal perfusion, the autonomic nervous system generates afferent arteriolar vasoconstriction in an attempt to maintain adequate glomerular pressure. This condition leads to reduced renal blood flow, ischemia, and reduced glomerular filtration [2]. Additionally, renal function is affected by reduced blood flow to the kidneys caused by mechanical compression of the abdominal vessels during laparoscopic pneumoperitoneum [2].

Alpha receptor agonists were introduced in anesthetic practice long ago. They modulate the sympathetic and endocrine metabolic response [4]. Dexmedetomidine (DEX) has both peripheral and central action, centrally inhibiting the locus coeruleus and the exocytosis of norepinephrine, leading to hypotension and bradycardia. The stimulation of α2-agonist receptors decreases sympathetic outflow, reducing circulating serum catecholamine levels, leading to a predominance of parasympathetic action and resulting in lower blood pressure [4].

There are no known absolute contraindications to the use of dexmedetomidine. Nevertheless, the most common adverse reactions to the use of DEX are hypotension (30%), hypertension (12%), nausea (11%), bradycardia (9%), and dry mouth (3%). The increase in DEX concentrations progressively decreases heart rate and cardiac output, and there is a potential for asystole, although rare [4].

DEX has been tested for several purposes, such as sedation in general anesthesia [5], prolonging the effect of peripheral blocks [6], postoperative delirium [7], reducing opioid consumption [5], reducing nausea and vomiting, and providing good control of hemodynamic response. Clonidine (an alpha-2 blocker) can prevent renal function deterioration in patients undergoing cardiac surgery [5]. More recently, there is evidence of the nephroprotective effect of continuous infusion of DEX during cardiac surgeries [6]. Also, DEX has benefits on overall surgery with general anesthesia in the overall population by reducing hospital mortality, ARI, and intensive care unit (ICU) LOS [8].

This research aims to systematically review the medical literature and trial registries regarding the effect of DEX on ARI, mortality, and LOS when used on elderly populations submitted to laparoscopy.

This article was previously posted to the medRxiv preprint server on May 14, 2025.

## Review

Materials and methods

This systematic review was conducted in accordance with the Preferred Reporting Items for Systematic Reviews and Meta-Analyses (PRISMA) 2020 guidelines and was registered in the International Prospective Register of Systematic Reviews (PROSPERO) under the ID CRD42023456529.

Eligibility Criteria

This study's target population was patients aged 60 years or older undergoing laparoscopic surgery. The intervention of interest was the use of DEX at any dosage, administered at anesthetic induction, during the surgical procedure, or after removal of the laparoscopic device. Placebo, other anesthetics, such as bupivacaine, and opioids were considered acceptable comparators. The outcomes assessed included hospital overall death, ARI defined by the author, urinary output (ml on the first day after surgery), and length of stay (LOS) in days. Only randomized clinical trials were included in the research design.

Search Strategy and Information Sources

The initial search strategy was performed in the bibliographic database PubMed, later adapted to Scopus, Web of Science, Cochrane trial databases, and Embase. The initial search strategy performed at PubMed was based on the health descriptors (NCBI MeSH) and the strategy was as follows: (laparoscop* OR videolaparoscop* OR "Laparoscopy"[Mesh]) AND ("Dexmedetomidine"[Mesh] OR precedex OR precedes OR dexmedetomidine) AND ("Elderly").

Selection Process and Data Collection Process

The abstracts identified from the remote search were assessed by the two blinded authors (YNK and PEAA) between March 2023 and March 2024. The abstracts were uploaded in Rayyan (https://www.rayyan.ai). These abstracts should meet at least partially the population, intervention, comparison, results and study design (PICOS) items. The disagreements were solved by consensus in weekly meetings and eventually by consulting the full manuscript. Later, the full manuscripts from all included abstracts were assessed, and their data were extracted to full forms in a blinded fashion. Disagreements were solved in consensus meetings. During the review, there was an attempt to match retrieved trial registries and full manuscripts filled out to join multiple sources from the same trial. E-mails were sent to authors in an attempt to retrieve unavailable data from both trial registries and full manuscripts' final reports. 

Data Items

Data collection research forms were developed and tested in several rounds. After being ready, they were inserted into REDCap (https://project-redcap.org) and structured with the sections: identification, eligibility, study characteristics, population characteristics, risk of bias, and outcome data.

The AKI concept of interest was initially defined as the Kidney Disease Improving Global Outcomes (KDIGO) criteria [9]. However, this concept was rare during the assessment of the original studies. Therefore, we accepted any definition of AKI in the original studies. Data presented in the manuscript but not in the expected format were requested from the authors. This includes LOS presented either as binary or as means and standard deviation when it was expected to be presented as medians, IQR (IQR), and range. Data was also requested when absent, for example, when in the methods section the authors mentioned that data would be analyzed but later stated it was not significant and not shown in tables or graphs, or when there was clear evidence that the sample had 60+ participants but no outcome data regarding this age strata was available. These e-mails were standardized, and the research form questions were pasted in the message body to facilitate a reply with the data of interest. 

Study Risk of Bias Assessment

Trials were evaluated with the Cochrane Risk of Bias tool version 2 (Rob2), of August 2019, and graded as "Low Risk", “High Risk”, and “Some Concerns", following the Rob2 documentation and instructions. In a manner similar to the classification steps, the two authors assessed the full manuscripts, extracted data while remaining blinded, and resolved any disagreements by reaching a consensus during weekly meetings. 

Effect Measures

For the outcome of overall hospital death and AKI incidence, the plan was to estimate the risk ratio; therefore, the number of participants with AKI and the total analyzed in the groups using DEX and the comparator were required. The same was performed regarding hospital mortality. For outcomes of urinary output and LOS, the plan was to estimate the standardized mean difference (SMD); therefore, when data of interest was reported as medians, it was transformed into means. 

Synthesis Methods

The synthesis was planned to include all clinical trials with available data. Therefore, the clinical trials with no data would be presented with zero weight in the meta-analysis. For the AKI and death outcomes, the inverse variance method was planned to estimate the pooled summary. For the urinary output and LOS, the standardized mean difference was used. Specifically for the LOS, in which the values distribution is known to be asymmetric, the standardized mean difference was planned to be a combination of reported means (and SDs) and means (and SDs) estimated from median and interquartile range and range using Cai’s method for unknown non-normal distributions (MLN) approach [10]. Pooled summaries were estimated with a common (fixed) effect model. Heterogeneity was assessed with the I² and Q heterogeneity test. Reporting bias was planned to be explored with a funnel plot and Egger test. All analyses were performed with meta and robvis packages in R project software (R Foundation for Statistical Computing, Vienna, Austria). 

Certainty Assessment

The results were tabulated and evaluated using the Grading of Recommendations Assessment, Development, and Evaluation (GRADE) method and the GRADEPro web application. For each GRADE dimension, the reviewers discussed how confident they were about the results and removed points from the dimension; later, the overall confidence is assembled by considering the confidence in each dimension. GRADE structures the recommendations as strong or weak based on the confidence of the body of evidence and the balance of benefits and harms [11,12].

Results

Study Selection 

The initial selection in database research returned 2,749 studies, and 652 replicates were removed, resulting in 2,097 abstracts that underwent screening by title and abstract, resulting in 45 studies included for full-text reading.

**Figure 1:**
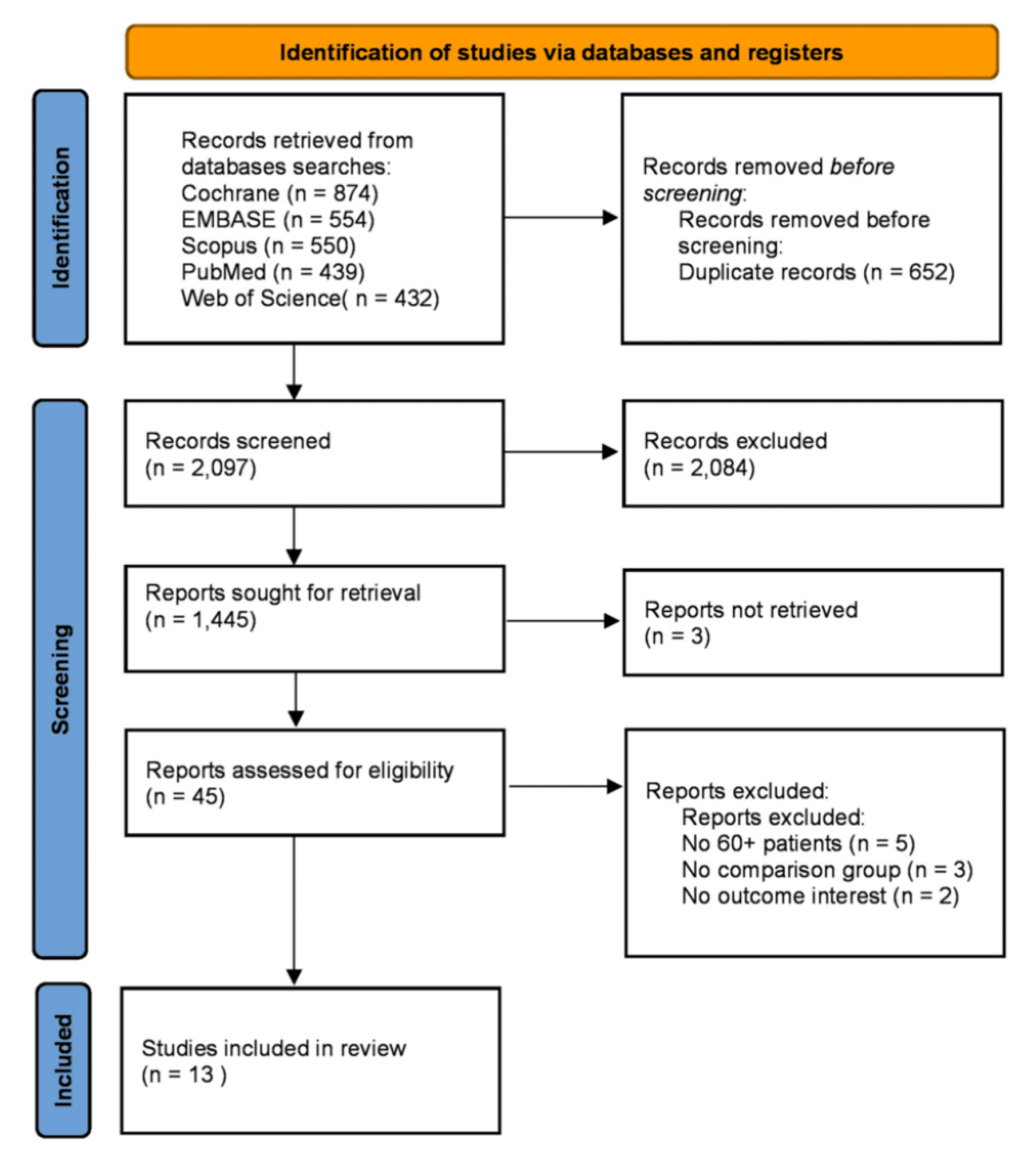
PRISMA inclusion and exclusion flow diagram of the study selection process. PRISMA, Preferred Reporting Items for Systematic Reviews and Meta-Analyses. n = sample size.

After abstract selection, 26 records were full manuscripts, and 19 were trial registries. Five studies didn't have patients aged 60+ in their sample, three studies didn't have a comparison group, and two studies didn't have any outcome of interest and were excluded. Two trial registries were replicates; only one trial registry was matched with a published manuscript. The remaining trial registries didn't have any available data on the registry website. No authors returned attempts to retrieve additional data regarding the trial registries or incomplete data from full manuscripts. In the end, there were 13 studies from which data could be extracted and analyzed (Figure 1).

Study Characteristics

Eleven reports were from China, one from India, and one from Ukraine (Table 1). All studies were published between 2016 and 2023. Although random allocation was always mentioned, the random allocation type wasn't clear in most studies, and the allocation concealment was not mentioned in almost half. Most trials mentioned double blinding, and it was always the participant and outcome measurement blinding. Many kinds of surgical clinics were involved (Table 1). The type of anesthesia varied according to the surgical proposal as follows: in eight studies, the approach was general anesthesia, in three was regional anesthesia, and one was infiltration of the portal site. The time and form of administration of DEX varied according to the anesthetic proposal and was used as an adjuvant in general anesthesia, regional anesthesia, or local anesthesia (Table 1). 

**Table 1:** Descriptive results of the included studies regarding study characteristics

ID	Country	Enrollment period	Random allocation	Allocation concealment	Blinding	Surgery	Type of anesthesia	Anesthesia timing
Wu-2019[13]	China	2014-2015	Ignored	Pharmacy control	Triple blind	Urological Surgery	General anesthesia	Induction/Continuous infusion
Sun-2021 [14]	China	2017-2018	Ignored	Sealed envelope	Triple blind	Colorectal Surgery	General anesthesia	Continuous infusion
Li-2023 [15]	China	2022-2023	Ignored	Sealed envelope	Double blind	Urological Surgery	Regional anesthesia	Induction
Chen-2016 [16]	China	2015-2015	Ignored	Ignored	Double blind	Colorectal Surgery	General anesthesia	Induction/Continuous infusion
Pan-2020 [17]	China	2017-2018	Ignored	Pharmacy control	Double blind	Colorectal Surgery	Regional anesthesia	Insufflation
Duan-2021 [18]	China	2019-2020	Ignored	Ignored	Double blind	Oncological Surgery	General anesthesia	Induction/Continuous infusion
Huang-2021 [19]	China	2016-2017	Ignored	Ignored	Double blind	Urological Surgery	General anesthesia	Induction/Post-surgery Continuous infusion
Yi-2022 [20]	China	2021-2022	Ignored	Ignored	Double blind	Oncological Surgery	General anesthesia	Induction/Continuous infusion
Bielka-2018 [21]	Ukraine	2016-2017	Block random	Ignored	Single blind	General Surgery	General anesthesia	Induction/Continuous infusion
Chen-2022 [22]	China	2018-2022	Ignored	Sealed envelope	Double blind	Oncological Surgery	General anesthesia	Induction/Continuous infusion
Jiang-2022 [23]	China	2014-2015	Ignored	Sealed envelope	Triple blind	Urological Surgery	General anesthesia	Others
Selvaraj-2019[24]	India	-	Ignored	Sealed envelope	Double blind	General Surgery	Others	Insufflation

The studies usually had small sample sizes, as usually the main outcomes were analogic scales of pain or analgesic rescue. Renal function, LOS, and hospital mortality were always secondary outcomes. The fraction of males was higher in most studies. Only one study included solely participants 60+ [10]; the remaining studies included a mix of participants in which there were participants at least 60 years old or younger in varying quantities. Although American Society of Anesthesiologists (ASA) classification was variable between the studies, patients classified as ASA II were the majority (Table 2). 

**Table 2:** Descriptive results of the included studies regarding study sample characteristics and outcomes ^a^Reported as a binary outcome, either longer or shorter than one day. ^b^Did not report AKI as a binary outcome. ^c^The single study included 60+ participants only. ^d^The comparator was bupivacaine. ^e^The comparator was ropivacaine. ID = identification; N = sample size; ASA = American Society of Anesthesiologists; AKI = Acute Kidney Injury; KDIGO = Kidney Disease Improving Global Outcomes.

ID	N	Fraction of males (%)	Age	Age central tendency	ASA I classification	ASA II classification	ASA III classification	Dexmedetomidine posology	Data for AKI?	AKI incidence	AKI criteria	Data for urine output?	Average urine output	Data for death?	Death incidence	Data for length of stay?	Median length of stay	Mean length of stay
Wu-2019^c ^[13]	89	100.00	NI	68.00	31.46	44.94	23.60	1μg/kg in 10 min + 0.5 μg/kg/h	Yes	8.99	KDIGO	Yes	1,508	No	-	Yes	12.50	-
Sun-2021 [14]	56	64.50	Average	59.50	-	-	-	1 μg/Kg + 0.5 μg/Kg/h	Yes	7.15	KDIGO	Yes	200	Yes	0	Yes	11.50	-
Li-2023^e ^[15]	55	61.18	Average	60.24	81.18	18.18	0.00	1 μg/kg	No	-	-	Yes	399	No	-	No	-	-
Chen-2016 [16]	60	48.35	Average	58.42	6.67	75.00	18.33	1μg/kg bolus + 0.3 μg/kg/h	No	-	-	No	-	No	-	Yes	-	8.92
Pan-2020 [17]	60	55.00	Average	60.85	0.00	70.00	30.00	0.5 μg/kg	No	-	-	Yes	609	No	-	Yes	-	9.35
Duan-2021 [18]	80	0.00	Average	70.01	-	-	-	0.5 μg/Kg in 10 min + 0.5 μg/Kg/h	No	-	-	No	-	No	-	Yes	-	5.66
Huang-2021 [19]	80	71.25	Average	52.50	21.25	78.75	0.00	0.5% 10min + 0.2 μg/Kg/h	No	-	-	No	-	No	-	Yes	-	8.49
Huang-2021 [19]	80	73.75	Average	52.24	20.00	78.75	0.00	0.5% 10min + 0.4 μg/Kg/h	No	-	-	No	-	No	-	Yes	-	8.51
Yi-2022 [20]	40	75.00	Average	52.00	0.00	65.00	35.00	0.5 μg/Kg 10 min + 0.5 μg/Kg/h	No	-	-	No	-	No	-	Yes	-	9.50
Bielka-2018 [21]	60	11.67	Median	54.00	-	-	0.00	0.5 μg/kg/h	No	-	-	No	-	No	-	Yes	3.04	-
Chen-2022 [22]	90	64.44	Median	55.75	0.00	74.40	25.56	0.3 μg/Kg/10 min + 0.3 μg/Kg/h	No	-	-	No	-	No	-	Yes	14.51	-
Jiang-2022 [23]	77	70.12	Average	52.04	-	-	0.00	0.6 μg/kg 10 min	Yes^b^	-	-	No	-	No	-	Yes	8.00	-
Selvaraj-2019^d ^[24]	116	33.00	Average	43.73	-	-	-	2mg/Kg	No	-	-	No	-	No	-	Yes^a^	-	-

Only three studies contained information on AKI, and its overall incidence ranged from 7.15 to 8.99 [13-15]. Two studies defined AKI based on the diagnostic criteria established by KDIGO. Four studies provided data on urinary output on the first day after surgery (Table 2).

Only one study provided data on hospital deaths [12]; on the other hand, only one study didn't provide data on the overall length of hospital stay [15]. No study provided the full distribution of median and IQR. Six studies provided mean LOS and SD [16-20], and five provided median LOS and its IQR [13,14,21-23]. One study provided LOS data as binary (Table 2) [15].

Most studies used placebo as a comparator, except for one using bupivacaine as a comparator [13] and one using ropivacaine as a comparator [22]. The authors used many kinds of administration ways and posologies, such as intravenous continuous infusions, in bolus, and as adjuvant of local infiltration, and the doses varied between 0.3 μg/kg up to 1 μg/kg, which were used through single shots or as continuous infusions.

Risk of Bias in Studies

The two dimensions that generated concerns of bias were the randomization process and deviations from the intended interventions. The issue regarding randomization is usually due to poorly described randomization procedures or concealment. The overall risk of bias assessment showed six studies with “Low Risk” of bias, 5 studies with “Some concerns,” and 1 study with “High Risk” of bias during the overall evaluation. 

Results of Individual Studies

**Figure 2:**
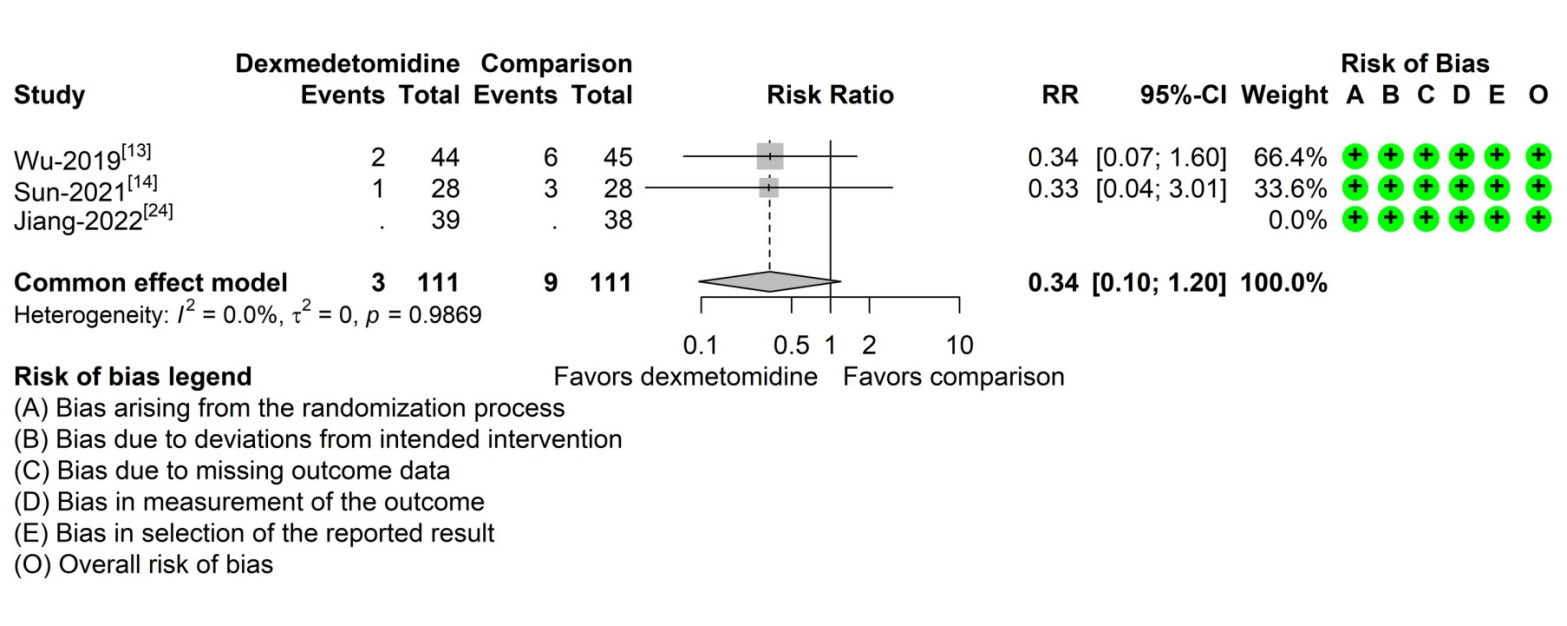
Forest plot of dexmedetomidine (DEX) vs. placebo effect on acute kidney injury

Out of the three studies reporting data regarding AKI [13,14,24,25], only two had sufficient data to perform a meta-analysis. All three studies had an overall risk of bias assessment as low risk. Both studies with risk ratio estimates had very similar effects favoring DEX benefits in preventing AKI, consistent in the same direction and with similar precision. With this data, heterogeneity was not detected. The imprecision of the pooled estimate is large; therefore, it seems there is a DEX potential benefit in preventing AKI, but more studies designed for this purpose are required to observe benefits with reasonable precision. Reporting bias was not explored due to the small number of studies (Figure 2).

**Figure 3:**
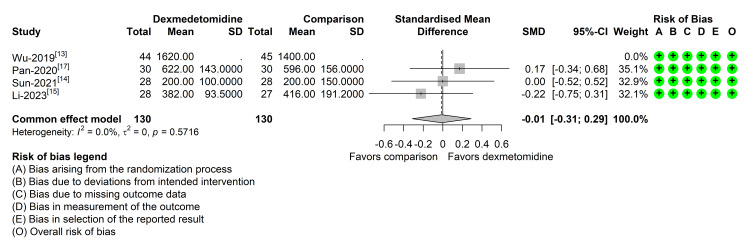
Forest plot of dexmedetomidine (DEX) vs. placebo effect on first-day urinary output SMD: standardized mean difference

Out of the four studies reporting data regarding urine output on the first admission day after surgery, one didn't have enough data to perform a meta-analysis [13]. Studies pointed to standardized mean differences in different directions, showing inconsistency in data. However, no study was able to detect a difference in urinary output among groups, and the pooled estimate also did not detect a significant effect. Heterogeneity was not detected with this data, and reporting bias was not explored due to the small number of studies. All studies included in this meta-analysis were classified as low risk of bias (Figure 3).

**Figure 4:**
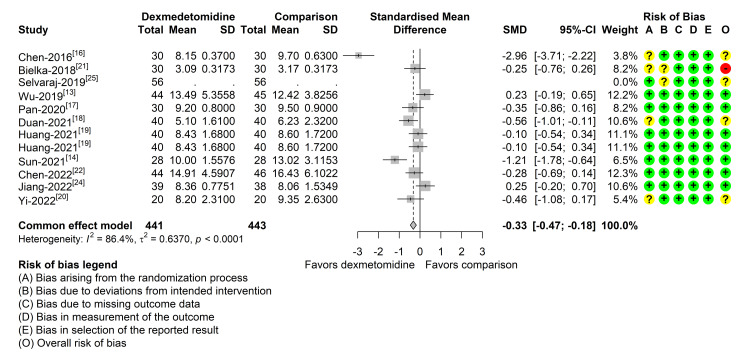
Forest plot of dexmedetomidine (DEX) vs. placebo effect on length of stay SMD: standardized mean difference

Out of the 12 studies reporting data regarding hospital LOS, one didn't have sufficient data to perform meta-analysis [26]. Most of the studies showed a neutral effect regarding LOS. Three studies detected a benefit favoring DEX in decreasing the LOS. However, two out of these three were the smallest studies included, and two of them had some concerns regarding the risk of bias assessment, which were the one with the largest difference and the one with the largest sample size out of these three. Despite the pooled estimate showing some effect favoring the use of DEX in reducing LOS, with less than a half-day difference, the magnitude of the effect is likely to be clinically not relevant. Additionally, there is a large evidence of heterogeneity, and out of the 12 studies, five had either some concern or high risk classification in the bias assessment (Figure 4). When performing the very same analysis and selecting the seven studies with an overall classification of low risk of bias, the heterogeneity reduces about 15%, yet very high, and the pooled estimate SMD moves to a neutral effect, not favoring DEX nor the comparison (data not shown). Additionally, in this subset, the smallest study with a sample size of 54 is the most influential and the one that most contributes to heterogeneity (data not shown).

Reporting Biases

During the review process, we came across the absence of specific data for the elderly population. Only one article contained data of patients aged 60+, and all others that included this population didn't separate the data specifically for elderly patients. We attempted to contact the authors by email to obtain specific data for the population 60+, but we didn't receive any response from any of the authors whose manuscripts were included in this review. 

We found a considerable number of trial registries with the potential to be included. However, none had available data on the registry website, even though many were way over the anticipated date to finish the trial.

There is also an issue regarding LOS reporting, as some manuscripts reported it as means and SD and others as median and IQR. Usually, LOS has an asymmetrical distribution and is skewed to the left. As the meta-analysis approach requires means and SD, in this scenario the data in the median and IQR were transformed into means and SD. However, without the range information, the means and SD estimates are more susceptible to bias. 

**Figure 5:**
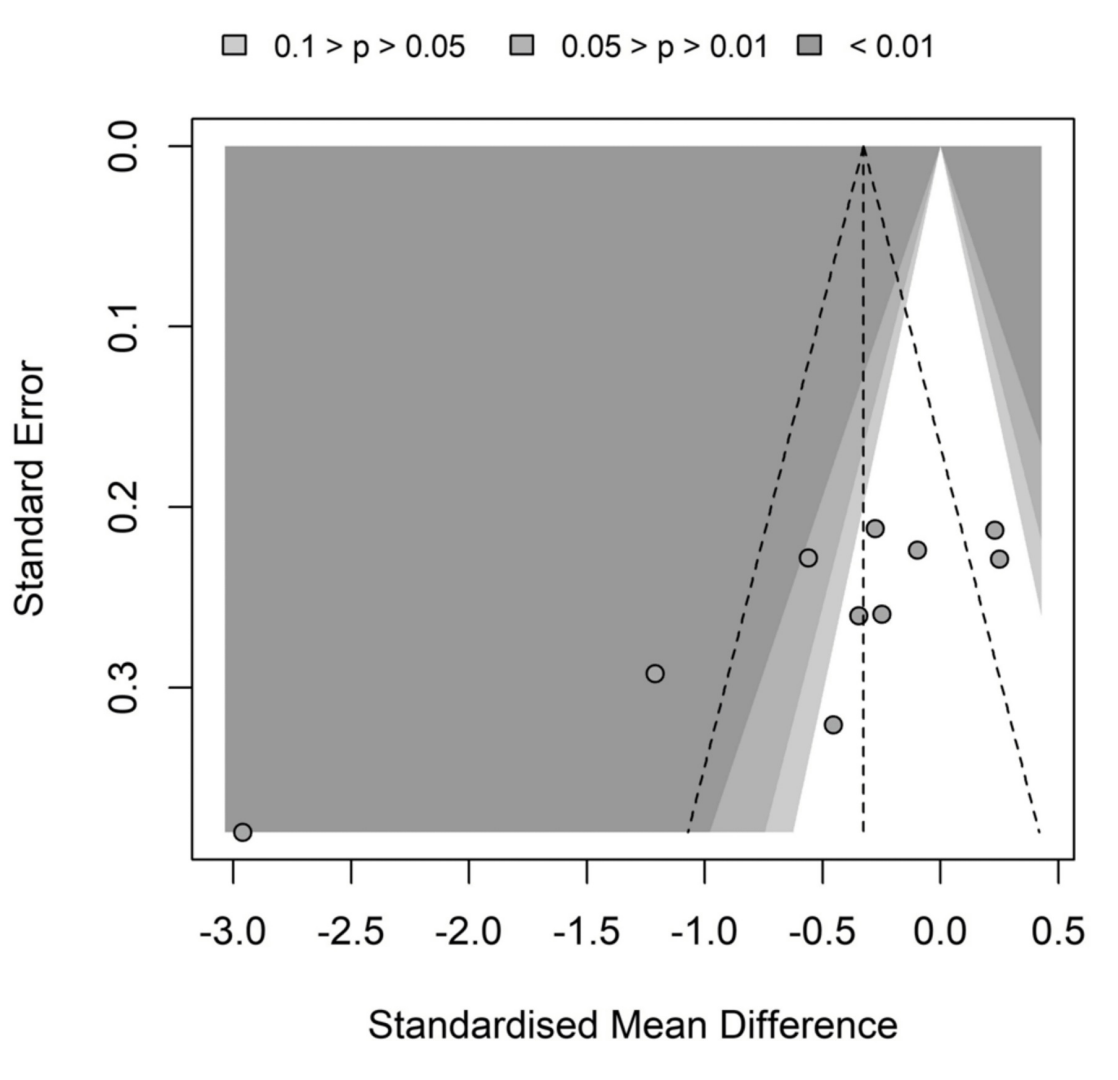
Funnel plot of dexmedetomidine (DEX) vs placebo effect on the length of stay (LOS) outcome

Despite there being two studies outside the funnel at each side, there is no visually evident asymmetry in the funnel plot of DEX effect on LOS (Figure 5). Nevertheless, the linear regression test of funnel plot asymmetry returned a very significant p-value of 0.0023, therefore, there are plenty of arguments and evidence for the presence of reporting bias.

Certainty of Evidence

A moderate level of confidence was found for the potential nephroprotective effect of DEX (as part of multimodal anesthesia) considering the body of evidence in studies including the elderly population undergoing laparoscopic surgery for radical prostatectomy and colorectal cancer who received DEX as part of the multimodal strategy in their anesthesia. There is plenty of evidence showing DEX has a beneficial effect on AKI incidence in other populations and other kinds of surgeries, which makes this potential benefit very plausible. The main issues reducing confidence in this finding are precision, as sample sizes were not planned to detect the benefit on AKI, and indirectness, as the effect on the elderly population is possibly mixed with the effect of the younger age group.

The confidence of the findings regarding the effect of DEX on reducing LOS is very low. The problems are in all dimensions of GRADE assessment. Additionally, even if there is a true beneficial effect of DEX in reducing LOS, the current body of evidence points to it being very unlikely to be clinically relevant, with a potential benefit of less than a third of a day than the comparison. Regarding urinary output, the confidence of findings given the current evidence is low. All GRADE dimensions for this outcome, but the risk of bias, were downgraded. Similar to LOS, the current body of evidence points to no beneficial effect of DEX on urinary output. 

Discussion

The main results to be discussed are: a) there is no evidence that intraoperative DEX in laparoscopic surgery reduces hospital LOS in the elderly; b) there is no evidence that intraoperative DEX in laparoscopy improves urinary output on the first day of admission in the elderly; c) there is evidence of the potential nephroprotective effect of intraoperative DEX in laparoscopic surgery in the elderly. If the nephroprotector effect is true, it seems it is not related to urinary output.

A reduction in length of hospital stay, delirium, time on mechanical ventilation, and ICU LOS was associated with DEX use in hospitalized patients; however, results appear to differ depending on the population and surgical setting [27-30]. To date, no studies on the elderly population have demonstrated a direct effect on reducing LOS, despite reports that it has a positive effect in the elderly population due to its anti-inflammatory action. This may be related to the pharmacological properties of the drug or possibly the timing and manner in which DEX is administered [27,31-33].

Studies have shown that laparoscopy is associated with decreases in LOS, ranging from 24 to 120 hours. Notably, colorectal resections approached laparoscopically have shown more pronounced benefits in the elderly compared to younger people, including lower morbidity rates and reduced LOS [34-36].

Once statistical heterogeneity is found regarding the effect of DEX on LOS, one may consider clinical or methodological sources for this heterogeneity. Even after removing studies with some concerns or high-risk bias, there is still some heterogeneity. Likely, differences among populations, DEX doses, and the diversity of clinics contributed to the heterogeneity. Not at random, missing data may also lead to heterogeneity. 

The quantification of urinary output involves maintaining an indwelling urinary catheter, which, according to studies, should be removed around 24-48 hours [37,38]. Although there are changes in urinary patterns related to age, elderly people maintain their urinary and renal function without changes if they receive adequate volume replacement in the perioperative period [39].

Studies have shown an increase in urinary output despite the use of DEX in various situations, both clinical and experimental. The mechanism behind this effect is believed to be related to blocking the release of antidiuretic hormone [40-42].

The measurement of urinary output is commonly associated with renal function. It is known that in the perioperative period, several factors influence urinary output, such as the release of vasopressin and aldosterone in response to surgical stimulus. Studies have shown that urinary output is reduced in patients under general anesthesia, making urinary output an unreliable marker of renal function in this setting [2,43,44].

The body of evidence in this review showed a neutral effect of DEX on urinary output. The studies analyzed show results in different directions, and the combined analysis didn't find a significant effect in any intervention [14,15,17]. Although this review found that DEX may have a nephroprotective effect in the studied population, this effect is not followed by an increase in urine output. Instead, it may be assessed by novel biomarkers of injury ARI.

Risk factors for postoperative AKI include advanced age, length of operation, and kind of surgery. AKI may also develop more quickly in the postoperative phase if there are concomitant risk factors [44]. In the process of AKI, changes in serum creatinine and urea are late markers of the ongoing injury. Using urea and creatinine to diagnose AKI may delay diagnosis and appropriate treatment. The adoption of novel biomarkers of acute kidney damage, such as Cystatin-C and neutrophil gelatinase-associated lipocalin (NGAL), may help in the early and conclusive diagnosis of AKI [2].

There is evidence pointing to DEX reducing AKI and enhancing renal function in the postoperative phase of non-cardiac procedures [44]. From the studies included in this review regarding AKI, it can be seen that there is no heterogeneity, and there is a non-significant but consistent potential nephroprotective effect. In this way, the use of DEX as an adjuvant in a multimodal strategy in the elderly has a potential benefit to reduce AKI and its consequences in this group of patients during the hospitalization period.

All reports included in this review didn’t perfectly match the review’s question, the population, and there are also variations regarding comparison and outcomes of interest, which often led us to encounter a lack of specific data addressing the original research question. During the analysis, we observed different dosages, routes of administration, and surgical times for the administration of DEX, which varied according to the researcher's interest in their primary objectives. Therefore, there is a degree of indirectness in the review analysis, especially regarding population and intervention definitions. The studies that allowed the evaluation of ARI and urinary output are few, six in total. They all had small sample sizes for this purpose. In addition, there is a lack of clarity regarding the definition of these outcomes as compared to the originally stated primary outcomes. It was not possible to explore reporting bias for all review outcomes, as for two of them the amount of included studies was very low for this purpose.

## Conclusions

We conclude that, based on the current data, DEX use in laparoscopies among the elderly didn’t impact the reduction of hospital LOS and shouldn’t be recommended for this purpose in this population. Similarly, DEX use in laparoscopies among the elderly didn't improve the urinary output and shouldn't be recommended for this purpose in this population. There is moderate confidence that DEX use in laparoscopies among the elderly has the potential to reduce AKI. To increase certainty regarding this conclusion, studies planned to use DEX as an intervention in laparoscopies among the elderly should take AKI as the primary outcome and should either include the elderly population exclusively or report results for elderly strata. AKI planned as the primary outcome will ensure that a reasonable sample size will be estimated in advance, and biomarkers to represent this outcome properly will be reasonably planned. 

DEX is already regularly used in multimodal anesthesia. There is no evidence that a particular dosage in this strategy is either beneficial or may do harm in this setting for this population in preventing AKI. However, likely, similar doses to those used to reduce opioid consumption, prevent postoperative nausea and vomiting (PONV), reduce shivering, and control hemodynamic parameters will be used to investigate AKI prevention.
